# Modeling Early Gambling Behavior Using Indicators from Online Lottery Gambling Tracking Data: Longitudinal Analysis

**DOI:** 10.2196/17675

**Published:** 2020-08-12

**Authors:** Gaëlle Challet-Bouju, Jean-Benoit Hardouin, Elsa Thiabaud, Anaïs Saillard, Yann Donnio, Marie Grall-Bronnec, Bastien Perrot

**Affiliations:** 1 Addictology and Psychiatry Department Centre Hospitalier Universitaire de Nantes Nantes France; 2 SPHERE INSERM UMR1246 University of Nantes, University of Tours Nantes France; 3 Biostatistics and Methodology Unit, Department of Clinical Research and Innovation Centre Hospitalier Universitaire de Nantes Nantes France

**Keywords:** gambling, internet, trajectory, latent class analysis, growth mixture modeling, gambling tracking data, early detection

## Abstract

**Background:**

Individuals who gamble online may be at risk of gambling excessively, but internet gambling also provides a unique opportunity to monitor gambling behavior in real environments which may allow intervention for those who encounter difficulties.

**Objective:**

The objective of this study was to model the early gambling trajectories of individuals who play online lottery.

**Methods:**

Anonymized gambling‐related records of the initial 6 months of 1152 clients of the French national lottery who created their internet gambling accounts between September 2015 and February 2016 were analyzed using a two-step approach that combined growth mixture modeling and latent class analysis. The analysis was based upon behavior indicators of gambling activity (money wagered and number of gambling days) and indicators of gambling problems (breadth of involvement and chasing). Profiles were described based upon the probabilities of following the trajectories that were identified for the four indicators, and upon several covariates (age, gender, deposits, type of play, net losses, voluntary self-exclusion, and Playscan classification—a responsible gambling tool that provides each player with a risk assessment: green for low risk, orange for medium risk and red for high risk). Net losses, voluntary self-exclusion, and Playscan classification were used as external verification of problem gambling.

**Results:**

We identified 5 distinct profiles of online lottery gambling. Classes 1 (56.8%), 2 (14.8%) and 3 (13.9%) were characterized by low to medium gambling activity and low values for markers of problem gambling. They displayed low net losses, did not use the voluntary self-exclusion measure, and were classified predominantly with green Playscan tags (range 90%-98%). Class 4 (9.7%) was characterized by medium to high gambling activity, played a higher breadth of game types (range 1-6), and had zero to few chasing episodes. They had high net losses but were classified with green (66%) or orange (25%) Playscan tags and did not use the voluntary self-exclusion measure. Class 5 (4.8%) was characterized by medium to very high gambling activity, played a higher breadth of game types (range 1-17), and had a high number of chasing episodes (range 0-5). They experienced the highest net losses, the highest proportion of orange (32%) and red (39%) tags within the Playscan classification system and represented the only class in which voluntary self-exclusion was present.

**Conclusions:**

Classes 1, 2, 3 may be considered to represent recreational gambling. Class 4 had higher gambling activity and higher breadth of involvement and may be representative of players at risk for future gambling problems. Class 5 stood out in terms of much higher gambling activity and breadth of involvement, and the presence of chasing behavior. Individuals in classes 4 and 5 may benefit from early preventive measures.

## Introduction

The prevalence of past-year gambling problems varies from 0.1% to 5.8% worldwide [1]. In France, it was estimated at 2.7% of the general population between 15 and 75 years old [2]. This prevalence reached 22.4% among the population of individuals who gambled online in 2017 [3], compared to 17.0% in 2012 [4]. Internet gambling has intrinsic features that may facilitate excessive gambling, such as high accessibility, anonymity, high frequency of gambling outcomes, and digital payment modes [5-7]. Individuals who gamble online have a higher risk and a higher severity of gambling problems [8-12].

Online gambling also provides a unique opportunity to monitor gambling behavior in real environments [13]. The larger part of gambling research has been performed using questionnaire-reported subjective data, but self-reported data has been widely criticized (even beyond the framework of gambling) because it lacks both accuracy and validity and is prone to numerous biases [14-17]. It has been emphasized that too little research has been conducted in a real gambling environment with individuals who gamble [18,19]. Thus, a stream of gambling-related research has been undertaken using of gambling tracking data (naturalistic data) [20]. Large research programs [21-36] have been initiated including research [36] that focuses on rarely studied online lottery gambling tracking data (lottery draws, daily lotteries, and scratch cards). Indeed, such gambling is traditionally considered the least associated with gambling problems, but is the most prevalent form of gambling, leading to an overall sum of low-level harms that could be as important as the harm associated with more problematic but less prevalent forms of gambling [37].

Gambling tracking data allow researchers to easily access activity such as money wagered, number of gambling days, deposits, wins, and losses. Such indicators are informative, but do not, on their own, identify the potential for future gambling problems (since individuals who engage heavily in gambling are not necessarily those who develop gambling problems). Consequently, the combination of activity-based indicators with indicators that are related to core features of addiction may better capture individuals at risk for gambling problems. One potential indicator of addiction is chasing behavior which is defined as the continuation or intensification of gambling after a sequence of losses with the objective to recover previous losses [38]. This behavior is almost omnipresent in individuals with gambling problems and has been identified as the most significant step in the development of pathological gambling [38-40]. It is considered a key indicator of problem gambling behavior, especially in research that uses gambling tracking data [20,41]. The identification of chasing episodes may be performed in either a between- (over a long timescale spanning multiple sessions) or within-session (bet-by-bet behavior) dimension [38]; however, the latter appears to be better at capturing chasing behavior [20]. Researchers often use the modification of betting behavior depending on previous betting outcomes as a proxy to identify chasing episodes, as it is a behavior that is not directly observable [20]. In recent work [36,42], we proposed to approximate within-session chasing behavior by focusing on recurrent deposits within a short period of time or deposits that occurred immediately after a bet. When a deposit is made in these conditions, it likely means that the person has recently lost money; the deposit indicates the unplanned continuation of gambling in an attempt to recover losses. Another potential indicator of problem gambling is the breadth of involvement, which is generally defined as the number of different games played by an individual [30] and is considered a form of variability in gambling [20]. It has been found to be higher for those who gamble online compared to those who gamble offline [12,30,43] and could be a mediator in the relationship between online gambling and gambling problems [44].

Rather than using cross-sectional data, a longitudinal approach may be more relevant to identify individuals at risk for gambling problems. Gambling activity (frequency and intensity) and gambling variability (daily variability and trajectory, ie, the increasing or decreasing pattern of wagers) have been used to monitor gambling patterns in live-action sports betting by individuals during the first month after their gambling account is created and to determine the association of patterns with the development of later gambling problems [23]. The results indicated that individuals with high-activity and high-variability gambling were more likely to close their account due to gambling-related problems. Moreover, in previous work [36], we found that risky monthly behaviors were associated with larger deviations from the usual gambling activity in online lotteries. Finally, in a recent review, trajectory information has been reported as a possible method with good predictive performance in identifying problem gambling behavior through online gambling tracking data analysis [20].

In this study, the early gambling trajectories of online lottery gambling during the first month after the creation of an account is modeled. The objective was to identify distinct profiles of individuals who gamble online which can be distinguished by their early trajectories and to characterize their patterns in relation to their potential for gambling problems. This work is part of the first stage of EDEIN (*Etude de Dépistage des comportements Excessifs de jeu sur Internet*; *Screening for Excessive Gambling Behaviors on the Internet*) [42] (ClinicalTrials.gov NCT02415296; http://clinicaltrials.gov/ct2/show/NCT02415296).

## Methods

### Participants

An anonymized data set was used; this data set had also been used in a previous analysis [36]. The data were comprised of gambling‐related records from a random sample of 10,000 clients of the French national lottery and included the age, gender, and Playscan classification—a responsible gambling tool that provides a low, medium, or high risk assessment—of each client. This operator is the only one in France that is permitted to offer online lotteries and scratch games, and thus, represents 100% of the market for online lotteries and scratch games in France [36]. The Playscan classification is a risk assessment based on an individual’s 5 previous weeks of gambling activity: green corresponds to a low risk of problem gambling, orange corresponds to a medium risk, and red corresponds to a high risk [45]. It uses a combination of quantitative (wagers, deposits, gambling session duration, etc) and qualitative (gambling in risky periods, etc) gambling behavior data to estimate risk of problem gambling. The initial dataset (n=10,000) included individuals who gambled at least once between September 2015 and August 2016. In this study, we were interested in early behaviors only; therefore, we restricted inclusion to individuals who created their account between September 2015 and February 2016 (n=1152). As a consequence, it was possible to observe gambling activity during the six months following account creation. We chose a period of six months in order to observe early gambling behaviors without focusing solely on the first few weeks, which are not necessarily representative of future gambling activity.

### Data Reduction

To observe changes in early gambling trajectories, each variable was computed based on a 15-day unit of time, starting with *t*=*t_0_* on the day of account creation, resulting in 12 equidistant time points (*t*=*t_0_*, *t_1_*, *t_2_*,…, *t_11_*) over the 6-month period for each newly registered individual.

To conduct the trajectory analysis, 4 measures were selected from the original data set and calculated for each 15-day unit of time: amount wagered (the sum of all the bets made), number of gambling days (the total number of days with at least one gambling session), the number of chasing episodes (the number of times that money was deposited into the gambling account was used as a proxy of chasing behavior and applicable only if criteria were met—deposits either 3 or more times within a 12-hour period or less than 1 hour after a bet) [36,42], and involvement (the number of different games played). The amount wagered and number of gambling days were used as indicators of gambling activity, while the number of chasing episodes and involvement were used as indicators of at-risk gambling behaviors.

In addition, we used the following covariates to characterize the profiles that were identified: gender, age, cumulative deposits over the 6-month period, largest single-day deposit during the 6-month period, percentage of bets on instant lotteries (scratch cards and instant draws) over the 6-month period, cumulative net loss over the 6-month period (calculated by subtracting winnings and promotional e-credits from wagers), voluntary self-exclusion during the 6-month period (a categorical variable defined as either yes, if there was at least one episode of self-exclusion, or no, if there were zero episodes of self-exclusion during the 6-month period), and the individual’s highest Playscan classification during the 6-month period. Net loss, voluntary self-exclusion, and Playscan classification were chosen to serve as external verification of problem gambling [20]. All amounts were recorded in euros and have been converted to US dollars (a currency exchange rate of €1=US $1.084 was applicable at the time of publication).

### Statistical Analysis

We used a two-step approach to establish typologies that group individuals who evolve differently over time (individuals with similar trajectories identified for the 4 indicators) using data from the initial six months of their online gambling subscription.

The first step of the analysis consisted of identifying trajectories for each of the 4 gambling indicators. To model the evolution of each indicator (measured at 12 discrete time points for each individual, in a highly heterogeneous population [46]), we used growth mixture models [47,48]. Models were selected based on statistical criteria and upon interpretation of the classes. For each indicator, we tested multiple models to determine the best number of trajectories (from 1 to 8). For each number of trajectories, we computed several models, starting with the most complex (linear, quadratic, and cubic time-dependent terms, an intercept term, and random effects on both intercept and slopes), which was then simplified, if necessary, based on statistical criteria (convergence and stability of the model, Bayesian information criterion, significance of the parameters). For each model, we randomly generated 400 sets of initial values, for which 10 iterations of the expectation-maximization algorithm were performed. For the 50 best solutions, the entire expectation-maximization algorithm was performed. A model was considered to be stable if the best log-likelihood was replicated (ie, if at least two solutions had the same final best log-likelihood). The selected trajectory models were compared with one another to determine the best model for each indicator. The outcomes of the growth mixture models were membership probabilities (the probability that an individual belonged to each modeled trajectory).

The second step was to perform classification by grouping individuals with similar indicator trajectories. A latent class analysis was performed using the trajectory membership probabilities as the observed variable. This strategy allowed us to define latent classes with distinct characteristics of gambling activity and at-risk behavior. Model selection was based on a trade‐off between the Bayesian information criterion, the classification error rate (which reflects the precision of the classification), the interpretability of the classes, and the replicability of the model. The classes were described by the trajectory membership probabilities of each indicator and by the covariates.

Such a two-step analysis strategy has been previously used in studies [49,50] in various health areas, including behavioral addictions. Growth mixture models were estimated using Mplus software (version 8.1; Muthen and Muthen) [51]. Latent class analysis was conducted using Latent Gold software (Statistical Innovations Inc) [52].

### Ethics

This study was approved by the research ethics committee (*Groupe Nantais d'Ethique dans le Domaine de la Santé*) on March 25, 2015. Because of the retrospective and noninterventional design of this study, consent of the individuals whose data were used was deemed unnecessary.

## Results

### Characteristics

The characteristics of the sample, which was composed of 1152 individuals who had newly registered for an online gambling account, are described in Table 1. The sample was mainly composed of men (male: 740/1152, 64.2%; female: 412/1152, 35.8%) and the sample had a mean of 39.83 (SD 12.65) years of age. Table 1 shows that the indicators of gambling activity varied highly both between individuals (between-subject SD) and over the 6-month period for a given individual (within-subject SD).

**Table 1:** Demographic and gambling characteristics of individuals who had newly registered for an online gambling account (over a period of 6 months).

Characteristics			Individuals (N=1152) n (%)	Mean^a^	SD	SD, between-subject^b^	SD, within-subject^c^	Minimum	Maximum
Age, years			—	39.83	12.65	N/A^d^	N/A	19	81
**Gender**									
	Male		740 (64.2)	—	—	—	—	—	—
	Female		412 (35.8)	—	—	—	—	—	—
**Gambling activity**									
	Money wagered, €^e^		—	16.98	68.83	52.82	44.15	0.00	1918.00
	Gambling days, n		—	1.27	2	1.48	1.36	0	15
	Chasing, n		—	0.10	0.85	0.55	0.65	0	26
	Involvement, n		—	1.15	2.23	1.5	1.66	0	31
	Deposits, €		—	10.46	35.29	25.98	23.89	0.00	835.00
	Largest single-day deposit, €		—	7.32	17.69	10.44	14.29	0.00	500.00
	Losses, €		—	4.43	417.85	120.8	400	–48748.40	712.40
	Instant lotteries, %		—	11	28	19	21	0	100
	Voluntary self-exclusion		6 (0.5)	N/A	N/A	N/A	N/A	N/A	N/A
	Playscan								
		Green	1032 (89.6)	N/A	N/A	N/A	N/A	N/A	N/A
		Orange	83 (7.2)	N/A	N/A	N/A	N/A	N/A	N/A
		Red	36 (3.1)	N/A	N/A	N/A	N/A	N/A	N/A

^a^For quantitative variables, data were averaged at a monthly level over the 6-month period. This was intended to be more representative and meaningful than the 15-day unit of time used for trajectory analyses.

^b^between SD: represents the fluctuations of monthly gambling activity between the individuals (between-subject standard deviation).

^c^within SD: represents the fluctuations of monthly gambling activity within the 6-month period for a given individual (within-subject standard deviation).

^d^N/A: not applicable.

^e^At the time of publication, a currency exchange rate of €1=US $1.084 was applicable.

### Growth Mixture Models

**Figure 1:**
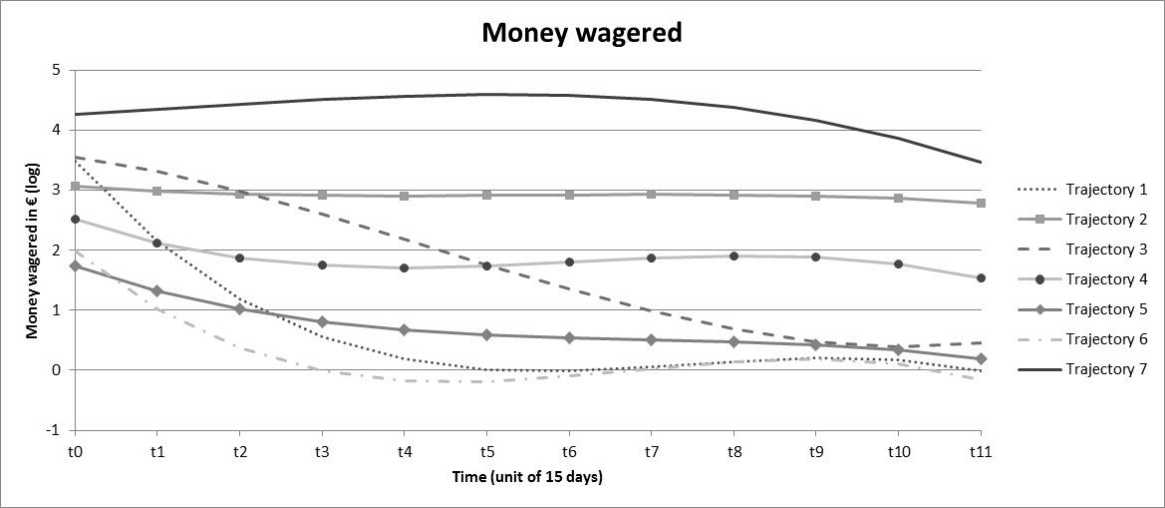
Trajectories obtained from growth mixture models for amount wagered. The ordinate axis represents the log-transformation of amount wagered. A value of 1 corresponds to €2, 2 to €6, 3 to €19, 4 to €54, and 5 to €147.

Seven trajectories were obtained for amount wagered and are shown in Figure 1. Trajectories 1 (13.0%), 3 (12.3%), and 6 (14.6%) exhibited a downward trend, initially showing a medium (trajectory 6) to high (trajectories 1 and 3) amount of money during initial weeks which gradually diminished to near zero amounts at the end of the 6-month period. Trajectories 2 (7.1%), 4 (26.4%), 5 (23.4%), and 7 (3.2%) were stable over the 6-month period. Trajectory 5 was characterized by low wagers—under €2 (US $2.17) per two weeks, trajectory 4 by medium wagers—€4-€12 (US $4.34-$13.01) per two weeks, trajectory 2 by high wagers—approximately €18 (US $19.51) per two weeks, and trajectory 7 by very high wagers—mainly €60-€100 (US $65.04-$108.40) per two weeks.

**Figure 2:**
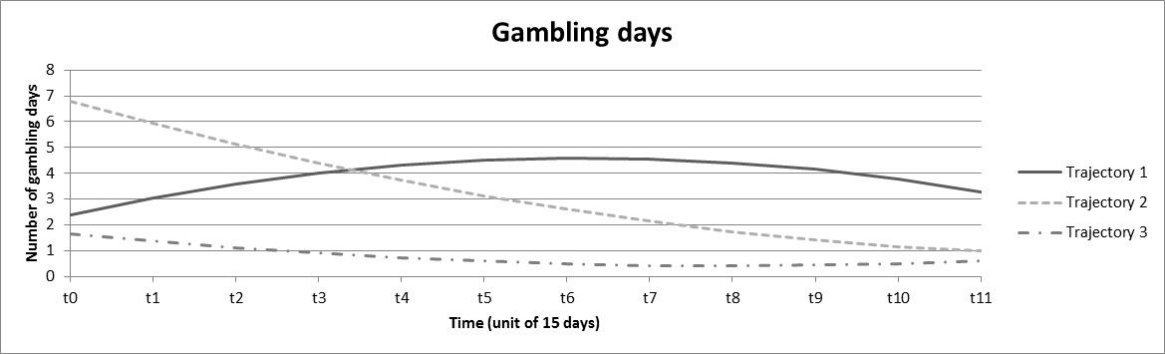
Trajectories obtained from growth mixture models for number of gambling days.

Three trajectories were obtained for the number of gambling days (Figure 2). Trajectory 1 (9.8%) was an inverted parabolic, increasing from 2.38 to 4.58 gambling days per two weeks, reaching a maximum at 120 days, and decreasing to reach 3.29 days at the end of the 6-month period. Trajectory 2 (9.8%) decreased rapidly from an initially high number of gambling days (6.8) which rapidly decreased to 0.98 days. Trajectory 3 represented the majority of individuals (83.4%) and had a relatively stable and low trajectory, with the number of gambling days fluctuating between 0.42 and 1.67.

**Figure 3:**
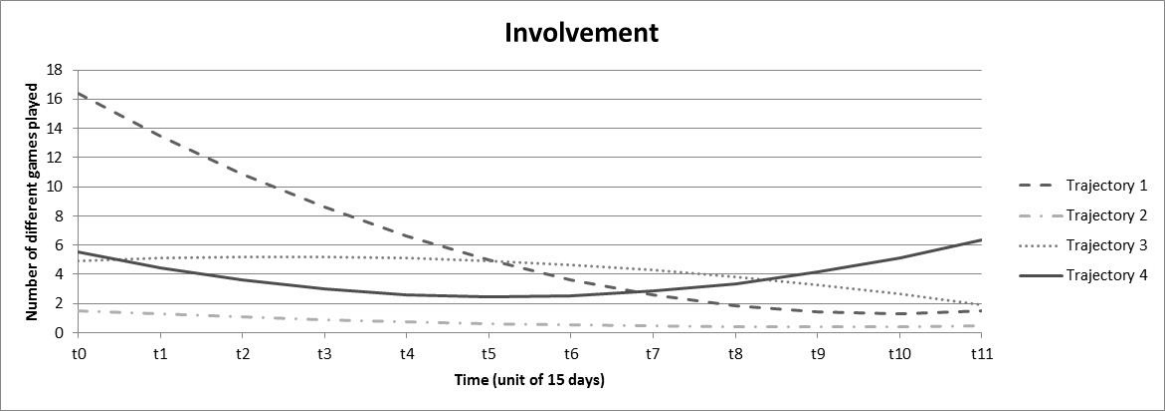
Trajectories obtained from growth mixture models for involvement.

Four trajectories were obtained for the involvement indicator (Figure 3). Trajectory 1 represented a very low proportion of individuals (1.3%), initially showing a very high diversity of games played (16.38 per two weeks) that rapidly decreased, and then stabilized to reach between 1.29-1.84 games after 120 days. Trajectory 2 represented the majority of individuals (89.9%) and was stable and low with approximately 1 (range 0.43-1.49) game played throughout the 6-month period. Trajectory 3 (5.8%) was also relatively stable, but with a higher number of games played, varying between 1.91 and 5.21. Finally, trajectory 4 (3.0%) exhibited a reverse parabolic shape, decreasing from 5.52 to 2.61 games played during the first 60 days, followed by a more pronounced rise after 90 days to reach 6.37 games at the end of the 6-month period.

**Figure 4:**
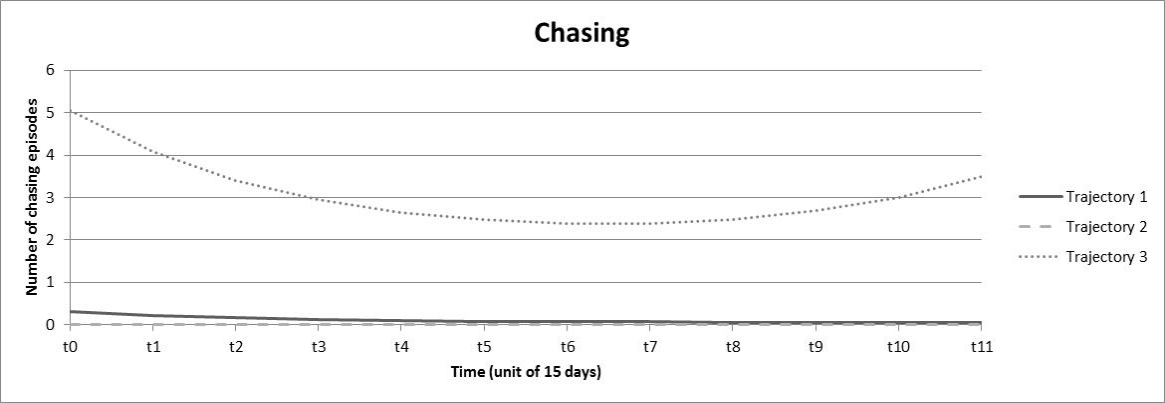
Trajectories obtained from growth mixture models for chasing.

Three trajectories were identified for chasing (Figure 4). Trajectories 1 and 2 were similar and represented the majority of individuals (trajectory 1: 33.9%; trajectory 2: 64.2%). They were characterized by the absence (trajectory 2: 0 episodes) or a very low number (trajectory 1: 0.06-0.31 episodes) of chasing episodes during the 6-month period. Trajectory 3 (1.9%) was an inverse parabolic and had a very high initial number (5.05 episodes per two weeks) which decreased to 2.39 episodes at 90 days and increased to 3.49 episodes at the end of the 6-month period.

### Latent Class Analysis

Fit indices of the models (1 to 8 classes) are given in Table 2. Bayesian information criteria decreased from the 1-class to the 8-class solution; however, the 7- and 8-class models were not stable. Because the 6-class solution did not yield a significant change in interpretation compared to that of the 5-class solution, the simplest model (ie, the 5-class model) was chosen.

**Table 2:** Fit indices of the 1- to 8-class models used to select the final model. A 5-class solution was selected.

Model type	Log-likelihood	Bayesian information criterion	Number of parameters	Classification errors
1-Class	–955	2150	34	0
2-Class	30077	–59667	69	0.0004
3-Class	38004	–75274	104	0.0006
4-Class	39901	–78823	139	0.0011
5-Class	43508	–85790	174	0.0016
6-Class	45205	–88937	209	0.0018
7-Class^a^	47402	–93085	244	0.0033
8-Class^a^	47837	–93707	279	0.0026

^a^Final log-likelihood not replicated.

Table 3 shows the distribution of the trajectories obtained for the gambling indicators and covariates for each class.

Class 1 (56.8%) was characterized by low gambling activity and low values for indicators of addiction; most individuals had the lowest trajectory of involvement and either a trajectory with no (*p*=.89) or few (*p*=.11) chasing episodes. Demographics of this class were similar to those of the overall sample (male: 64.8%; female: 35.2%; mean age: 39.06 years). Individuals from this class predominantly played deferred lotteries (79% of lotteries). They had low cumulative losses (€37.22) and deposits (€48.15) over the 6-month period. Almost all individuals (97.9%) were within the green Playscan classification.

Class 2 (14.8%) was characterized by medium to high and stable level of money wagered. For the number of gambling days, individuals in class 2 had either a low and stable trajectory (*p*=.61) or a medium and parabolic trajectory (*p*=.34). Moreover, they had similar indicators of addiction as those in class 1, but with a higher probability (proportion) for the trajectory that represented few chasing episodes (*p*=.59 compared to *p*=.11). The mean age of 43.41 years was higher than that of the overall sample and the proportion of women was lower than that in the overall sample (26.5%). These individuals were the only ones who had negative losses (ie, won €161.13), despite higher cumulative deposits (€154.97) than class 1. They predominantly played deferred lotteries (88% of lotteries) and were mainly (90.0%) within the green Playscan classification.

**Table 3:** Distribution of the trajectories for the gambling indicators and description of covariates among the 5 classes.

Model outcomes			Class 1	Class 2	Class 3	Class 4	Class 5
Probabilistic class size, in %			56.8	14.8	13.9	9.7	4.8
**Probabilities,** *p*							
	**Amount wagered**						
		Trajectory 1	.17^a^	<.001	.24^a^	<.001	.002
		Trajectory 2	<.001	.37^a^	<.001	.001	.33^a^
		Trajectory 3	.06	.02	.35^a^	.28^a^	.24^a^
		Trajectory 4	.20^a^	.57^a^	.08	.52^a^	.13^a^
		Trajectory 5	.33^a^	.03	.28^a^	.02	<.001
		Trajectory 6	.24^a^	<.001	.05	<.001	<.001
		Trajectory 7	<.001	.003	<.001	.18^a^	.30^a^
	**Gambling days**						
		Trajectory 1	.003	.34^a^	.004	.23^a^	.47^a^
		Trajectory 2	.001	.05	.16^a^	.25^a^	.31^a^
		Trajectory 3	.997^a^	.61^a^	.84^a^	.52^a^	.22^a^
	**Chasing**						
		Trajectory 1	.11^a^	.59^a^	.71^a^	.67^a^	.48^a^
		Trajectory 2	.89^a^	.41^a^	.29^a^	.33^a^	.13^a^
		Trajectory 3	<.001	<.001	<.001	<.001	.39^a^
	**Involvement**						
		Trajectory 1	<.001	<.001	<.001	<.001	.27^a^
		Trajectory 2	.999^a^	.998^a^	.994^a^	.39^a^	.15^a^
		Trajectory 3	.001	.002	.005	.41^a^	.35^a^
		Trajectory 4	<.001	.001	.001	.19^a^	.23^a^
**Covariates**							
	Age^b^, years		39.06	43.41	38.21	4.33	41.68
	**Gender, %^c^**						
		Male	64.8	73.5	62.1	54.9	52.0
		Female	35.2	26.5	37.9	45.1	48.0
	Voluntary self-exclusion, %^c^		0	0	0	0	10.8
	Cumulative losses^d^, €^e^		37.22	–161.13	80.85	189.65	541.64
	Cumulative deposits^d^, €^e^		48.15	154.97	102.91	232.99	797.05
	Largest single day deposit, €^e^		23.27	31.02	31.19	39.16	74.91
	Instant lotteries, %^c^		21	12	39	65	78
	**Playscan, %^c^**						
		Missing	0.5	0	0.6	0	0
		Green	97.9	90.0	9.6	66.4	28.7
		Orange	1.5	8.8	18.1	24.6	31.8
		Red	0.2	1.2	0.6	9.0	39.5

^a^These probabilities are the main trajectories represented within each class (*p*>.10).

^b^Values represent the mean for each class.

^c^Probability of belonging to each class. The percentages indicated refer to this probabilistic approach but do not represent a proportion of individuals.

^d^Cumulative over the 6-month period.

^e^At the time of publication, a currency exchange rate of €1=US $1.084 was applicable.

Class 3 (13.9%) was characterized by generally decreasing trends in gambling activity (amount wagered and the number of gambling days). Moreover, these individuals had similar indicators of addiction to those in class 2, but with a higher probability (proportion) for the trajectory that represented few chasing episodes (*p*=.71 compared to *p*=.59). Demographics of this class were similar to those of the overall sample (male: 62.1%; female: 37.9%; mean age: 38.21 years). These individuals displayed moderate cumulative losses (€80.85) and deposits (€102.91) over the 6-month period. They predominantly played deferred lotteries (61% of lotteries), but to a lesser extent than those in classes 1 and 2, and were mainly (90.6%) within the green Playscan classification.

Class 4 (9.7%) was characterized by medium to very high wagers, with the moderate and stable trajectory most represented (*p*=.52). For number of gambling days, individuals were distributed across the three trajectories with predominance in the low stable trajectory (*p*=.52). In contrast to classes 1, 2, and 3, this class was characterized by a combination of three trajectories for involvement with the majority in low and medium stable trajectories (*p*=.39 and *p*=.41, respectively). Moreover, individuals from class 4 had a similar pattern of chasing episodes as those from class 3. As a consequence, the pattern of class 4 was characterized by a diversification of games played but not by an increase in chasing episodes which remained relatively rare. The mean age of 40.33 years was similar to that of the overall sample and the proportion of women was higher than that in the overall sample (45.1%). These individuals displayed high cumulative losses (€189.65) and deposits (€232.99) over the 6-month period. In contrast to the first three classes, they predominantly played instant lotteries (65% of lotteries). The majority of individuals from this class were within the green Playscan classification (66.4%), but a significant proportion were within the orange (24.6%), and to a lesser extent, red (9.0%) classifications.

Class 5 (4.9%) was characterized by medium to high wagers, with the stable and high (*p*=.33) and the stable and very high (*p*=.30) trajectories most represented. Individuals from class 5 were also distributed in the three trajectories for the number of gambling days, but with a relative predominance of the high and parabolic trajectory (*p*=.47). Thus, individuals from this class had high or very high levels of gambling activity which remained high throughout the 6-month period. They were also characterized by a higher levels of involvement (all 4 trajectories) and were the only ones for which the highest-level trajectory of chasing was represented (*p*=.39). As a consequence, this class was characterized by both a diversification of games played and a large increase in chasing episodes. The mean age of 41.68 years was similar to that of the overall sample and the proportion of women was higher than that in the overall sample (48%). These individuals had very high cumulative losses (€541.64) and deposits (€797.05) over the 6-month period which were approximately 15 times more than the individuals in class 1 experienced and 3 times more than the individuals in class 4 experienced. They were also characterized by very high largest single-day deposits (€74.91) compared to those of the other classes. They predominantly played instant lotteries (78% of lotteries) and were mainly within orange (31.8%) and red (39.5%) Playscan classifications. Finally, this was the only class for which voluntary self-exclusions were present (10.8%).

## Discussion

### Principal Findings

The aim of this study was to investigate the early gambling trajectories of individuals over the initial 6 months of their subscription to the French online lottery website. We identified 5 distinct profiles of online lottery gambling, and we characterized each pattern in relation to indicators of gambling activity and gambling addiction.

The first three classes represented the majority of individuals (85.5%) and were characterized by low to medium gambling activity and low levels of problem gambling indicators. According to our assumptions, such individuals do not seem to encounter difficulty with their gambling practices and their gambling may be considered recreational. This was supported by findings of low losses (and sometimes wins), no voluntary self-exclusion, and low-risk Playscan classification (green tags).

In parallel, we identified 2 profiles that should be considered for early intervention or harm minimization. Class 4 represented approximately 10% of the sample and was characterized by medium to high gambling activity, a diversification of games played, and zero to few chasing episodes. The higher breadth of involvement may reflect more variable gambling activity and has previously been associated with account closure and classification of the individual as high risk by responsible gambling indicators [20]. Engagement in multiple online gambling activities has been identified as a potential predictor of high-risk gambling or the emergence of gambling problems [5]. Consequently, we can suggest that individuals in Class 4 may be at risk for future gambling problems but may not yet be gambling excessively. This assumption is supported by the fact that these individuals displayed high losses but were predominantly considered low and medium risk (green and orange, respectively) and did not use the voluntary self-exclusion measure. They played predominantly instant lotteries (65%), which have been found to be more associated with gambling problems than deferred lotteries are, due to high event frequency [7,36,53].

Class 5 was the smallest (4.8%) and was characterized by medium to very high gambling activity, a higher diversification of games played, and a high number of chasing episodes. Higher breadth of involvement and chasing behavior have been consistently associated with problem gambling [20]. Chasing, in particular, is considered a key indicator of problem gambling [20,41] and has been found to be the best discriminator between social and problem gambling among women [54]. Because class 5 was the only class with nonzero chasing trajectories, there is strong reason to believe that such gambling may be problematic. This is supported by the fact that these individuals experienced higher net losses and had the highest proportion among their class (71.3%) who were considered medium and high risk (orange and red tags). Moreover, they played forms of lottery that are more associated with gambling problems (ie, instant lotteries). More importantly, this class was the only class in which voluntary self-exclusion was present in a proportion (10.8%) that was higher than that observed in general online gambling (1%) [55], but close to that found in at-risk individuals who gamble online (11%) [56]. Voluntary self-exclusion is a harm-minimization strategy for individuals who experience gambling problems. In the case of online gambling, it consists of voluntarily banishing oneself from gambling websites for a predefined period [57]. Voluntary self-exclusion is considered a valid proxy indicator to externally verify which individuals have gambling problems [20], and thus, reinforced our deduction that individuals in class 5 may have gambling problems.

The whole sample was characterized by a higher proportion of women (35.8%) than that found in other studies using gambling tracking data from online gambling: 5.5% [29], 8% [23], and 10% [35]. Given that games of chance, such as lotteries, are more appealing to women than skill-based games are [58], this higher proportion was expected. This finding is also consistent with a French survey on the prevalence of online gambling [3] which found that 38.8% of the individuals involved in online lotteries were women; however, it was surprising that we found a higher proportion of women in the two classes exhibiting at-risk profiles. This was unexpected because it has generally been shown that men gamble more than women [1,59] and that at-risk individuals are predominantly men [60,61]. This unexpectedly higher proportion of women in at-risk classes was previously described in the same data set in a study that did not restrict inclusion to newly registered individuals [36]. It is worth noting that this tendency was maintained in early trajectories, making women a particularly vulnerable population for problem gambling when they initially open online accounts. As reported in Perrot et al [36], women with gambling problems may prefer to gamble online because they experience less stigma [6] and because they may be more socially anxious [62]. Moreover, boredom has been found to be both a motivating factor for gambling and a factor associated with continued problem gambling among women [58,63]. One can hypothesize that, beyond social anxiety and stigma, women with gambling problems may tend to choose online gambling to avoid boredom because it is accessible 24/7 and may represent a way to stay occupied to avoid negative emotional states.

### Limitations and Strengths

This study has several limitations. First, because we were not able to directly measure gambling problems, we were constrained to use proxies (net losses, Playscan status, and voluntary self-exclusion); however, these proxies have previously been used for external verification of gambling problems in gambling studies that use tracking data [20]. Otherwise, gambling problems have generally been measured using self-report questionnaires, such as in LaPlante et al [30], but self-reporting has limited representativeness given its low percentage of respondents [20]. Second, we only investigated a sample of individuals who play online lotteries. It would be interesting to replicate this work in other types of online gambling and will be the subject of future investigations; the results herein constitute only one part of the EDEIN research program and future EDEIN studies will address this limitation. Third, the choice of indicators for the trajectory analysis is questionable since other indicators exist; however, these choices had the advantage of combining information on gambling activity and on the potential for gambling problems. Fourth, the data set included only gambling activity related to online lotteries within a limited period. As a consequence, it is possible that some individuals in this study were also engaged in other types of gambling that were not captured by this study (other online or offline gambling activities) or that some individuals previously had an account on the same website which would bias the notion of describing “early” trajectories.

Despite these limitations, we must emphasize the strengths of this study. First, the naturalistic nature of gambling tracking data has high value for gambling research [13]. Second, we used a valid and robust method to explore trajectories whose utility has previously been demonstrated [49,50]. Trajectories as indicators for problem gambling have previously been defined using the slope of a linear regression that modeled wager size according to the sequence of active betting days during the initial month [23]. Such an approach may strongly reduce the information available because only positive or negative linear trends during the first month are observed and the following months, more complex trajectories, and other indicators of gambling are neglected. In our study, we used growth mixture modeling to model trajectories, which allowed us to capture the complexity of the evolution of gambling practice over time. Moreover, we performed this trajectory analysis on 4 indicators rather than only on wagers, which gave us access to the monetary variations over time as well as variations in gambling frequency, breadth of involvement, and chasing. Finally, the 6-month period allowed access to more in-depth trajectory information. Indeed, as shown in Figures 1 to 4, the variations of gambling activity during the first month did not necessarily reflect activity during the subsequent months. Third, Deng et al [20] emphasized that data aggregation often cannot capture chasing behavior, which requires fine-grained data. In our study, a chasing proxy was computed (based on the temporality between deposits and bets on a within-session basis) before data were aggregated. Because the chasing indicator best contributed to the identification of the problematic class, this definition may be useful for identifying individuals who are potentially at risk for gambling problems.

### Conclusion and Perspectives

We demonstrated the importance of using longitudinal trajectory models rather than cross-sectional analysis to identify groups of individuals who are potentially at risk for gambling problems. High breadth of involvement and high and sustained chasing, combined with the use of trajectory models, may be used early on to identify individuals who are at risk of experiencing gambling problems. More specifically, the probabilities of individuals belonging to the 5 classes identified can serve to implement personalized preventive actions. Indeed, the effectiveness of self-regulation strategies from responsible gambling programs, such as the setting of gambling limits for oneself or voluntary self-exclusion, may be limited by the fact that they completely rely on individuals changing their own behavior. As argued by Haefeli et al [64], it is of high importance to detect excessive gambling as early as possible before individuals reach the late stages of gambling problems and experience too much damage to be receptive to preventive interventions. The ability to rapidly identify individuals who are at risk for future gambling problems may allow the implementation of targeted, personalized minimal interventions. Such interventions could rely on valuing help-seeking, or on informing at-risk individuals about existing tools to prevent excessive gambling and possible gambling-related damage. This is the ultimate goal of the EDEIN project [42] of which this study was a first step.

## Abbreviations

EDEIN: Etude de Dépistage des comportements Excessifs de jeu sur Internet (Screening for Excessive Gambling Behaviors on the Internet)
